# Ferroportin depletes iron needed for cell cycle progression in head and neck squamous cell carcinoma

**DOI:** 10.3389/fonc.2022.1025434

**Published:** 2023-01-09

**Authors:** Benjamin Ross Belvin, Janina P. Lewis

**Affiliations:** ^1^ Philips Institute for Oral Health Research, School of Dentistry, Richmond, VA, United States; ^2^ Department of Biochemistry and Molecular Biology, School of Medicine, Richmond, VA, United States; ^3^ Department of Microbiology and Immunology, School of Medicine, Virginia Commonwealth University, Richmond, VA, United States

**Keywords:** iron/cell proliferation, ferroportin, head and neck squamous cell carcinoma, iron metabolism, cell proliferation, senescence, Oral Epithelial Cells

## Abstract

**Introduction:**

Ferroportin (FPN), the only identified eukaryotic iron efflux channel, plays an important role in iron homeostasis and is downregulated in many cancers. To determine if iron related pathways are important for Head and Neck Squamous Cell Carcinoma (HNSCC) progression and proliferation, we utilize a model of FPN over-expression to simulate iron depletion and probe associated molecular pathways.

**Methods:**

The state of iron related proteins and ferroptosis sensitivity was assessed in a panel of metastatic HNSCC cell lines. Stable, inducible expression of FPN was confirmed in the metastatic HNSCC lines HN12 and JHU-022 as well as the non-transformed normal oral keratinocyte (NOK) cell line and the effect of FPN mediated iron depletion was assessed in these cell lines.

**Results:**

HNSCC cells are sensitive to iron chelation and ferroptosis, but the non-transformed NOK cell line is not. We found that FPN expression inhibits HNSCC cell proliferation and colony formation but NOK cells are unaffected. Inhibition of cell proliferation is rescued by the addition of hepcidin. Decreases in proliferation are due to the disruption of iron homeostasis via loss of labile iron caused by elevated FPN levels. This in turn protects HNSCC cells from ferroptotic cell death. Expression of FPN induces DNA damage, activates p21, and reduces levels of cyclin proteins thereby inhibiting cell cycle progression of HNSCC cells, arresting cells in the S-phase. Induction of FPN severely inhibits Edu incorporation and increased β-galactosidase activity, indicating cells have entered senescence. Finally, in an oral orthotopic mouse xenograft model, FPN induction yields a significant decrease in tumor growth.

**Conclusions:**

Our results indicate that iron plays a role in HNSCC cell proliferation and growth and is important for cell cycle progression. Iron based interventional strategies such as ferroptosis or iron chelation may have potential therapeutic benefits in advanced HNSCC.

## Introduction

Head and neck squamous cell carcinoma (HNSCC) is the 6^th^ most common malignancy worldwide with more than 800,000 new cases and 450,000 deaths annually (1). Patients with recurrent or metastatic (R/M) HNSCC have a poor prognosis, with a median survival rate of under 1 year (2). Due to this, there is an ongoing effort to better understand the drivers of HNSCC progression and metastatic spread to develop novel and effective therapeutic strategies (3, 4). Iron has emerged as a key metabolic driver of malignancy, with many cancer types exhibiting an iron “addicted” phenotype.

The increased acquisition of iron in tumor cells has been shown to contribute to their growth and progression (5). Due to their increased proliferation, cancer cells have an elevated metabolic demand for iron when compared to normal cells (6). Consequently, genes related to iron homeostasis are commonly deregulated in many cancer types. Through alterations in cellular iron metabolism, cancer cells accumulate higher levels of labile iron through mechanisms involving increased iron uptake and retention as well as decreased iron efflux (7). However, our understanding of iron homeostasis in HNSCC is scarce.

It is noteworthy that eukaryotic cells utilize multiple mechanisms to import iron, but there is only one mechanism to export iron out of cells. This mechanism utilizes the iron efflux channel ferroportin (FPN) (8). Although it is of significant importance in many malignancies and iron-based disorders, the role of FPN in oral keratinocytes is yet to be determined. The repression of FPN expression has been demonstrated in multiple cancer types and provides a mechanism for the retention of cellular bio-available iron needed for cancer cell proliferation and cell cycle progression (9–11). However, this increased intra-cellular iron also makes cancer cells vulnerable to iron-based therapies such as ferroptosis and iron chelation (12, 13).

There is growing evidence that iron metabolism is altered in HNSCC. Mechanisms involving both an increase in iron uptake and retention have been observed. There is a noted increase in the levels of transferrin receptor 1 (TFR1) in clinical isolates of esophageal tumors (14). Furthermore, co-amplification of *TFRC* and *PIK3Ca* genes correlated with increased distant metastasis and poor prognosis in HNSCC patients (15). There is mounting evidence that the iron storage protein, ferritin (FTN), is significantly up-regulated in HNSCC and this increase corresponds to a poorer prognosis (16–18). Furthermore, increased levels of serum ferritin correlate with an increase in HNSCC lymph node metastasis (19). In a panel of HNSCC cell lines, homeostatic iron regulator protein (HFE) over-expression increased intra-cellular iron *via* an increase in hepcidin expression (20). Hepcidin binds directly to FPN, thereby causing its internalization and eventual degradation (21).

However, despite the evidence of deregulation of iron-related genes in HNSCC little is known about the molecular mechanisms behind the abnormality. Specifically, there are no studies focused on the impact FPN has on HNSCC progression. Even less is understood why HNSCC cells deregulate their iron homeostasis. Studies in other cancer types have implicated elevated iron levels accounting for increased activity of iron containing enzymes involved in DNA replication and nucleotide synthesis (22, 23). In this study, we modulate FPN levels to better understand the function of iron in advanced, metastatic HNSCC cell lines to understand how the loss of iron affects growth and proliferation in these cells. We reveal that FPN mediated iron efflux has substantial effects on HNSCC growth and is capable of inducing DNA damage, cell cycle arrest, and subsequent senescence in these cells but not in a non-malignant, TERT-immortalized normal oral keratinocyte (NOK) model. Ultimately, the expression of FPN negatively affects tumor growth and progression in a murine model of oral cancer.

## Results

### HNSCC cells exhibit increased iron dependence and are susceptible to ferroptosis

**Figure 1:**
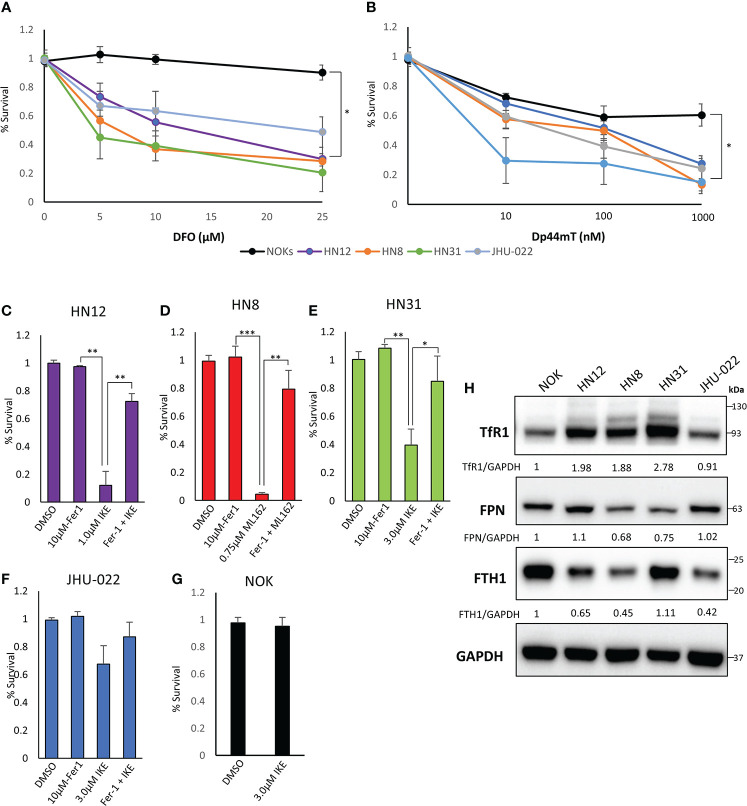
HNSCC cell lines exhibit an increased dependence on iron and are susceptible to iron chelation and ferroptosis. **(A)** Cells were grown with increasing amounts of deferoxamine (DFO) for 72 hours and cell viability was assessed *via* CellTiter-Glo assay. **(B)** Cells were treated with the indicated amount of Dp44mT for 72 hours and cell viability was measured *via* CellTiter-Glo. **(C–G)** The indicated cell line was seeded and treated with a vehicle control (DMSO), Ferrostatin-1 (Fer-1, 10μM), the indicated ferroptosis inducer (IKE or ML162), or a combination of both for 72 hours. Cell viability was measured *via* CellTiter-Glo assay. Values were normalized to a non-treated control with DMSO. **(H)** Western Blot of TFR1, FPN, FTH1, with GAPDH as a loading control. Quantification of TFR1, FPN, and FTH1 was performed using GAPDH as a loading control and normalized to the NOKs (*P<0.05; **P<0.01; ***P<0.001).

Iron-based therapies are emerging as a potential therapeutic strategy for the treatment of HNSCC. We tested the susceptibility of 4 HNSCC cell lines derived from metastatic sites to iron chelators DFO and Dp44mT (24, 25). As a non-malignant control, a TERT-immortalized non-transformed normal oral keratinocyte (NOK) cell line was used (**Figures 1A, B**). The HNSCC cells showed significantly higher sensitivity to iron chelation when compared to the non-transformed NOK cell line.

Next we tested the susceptibility of these cell lines to ferroptosis inducers imidazole ketone erastin (IKE) and ML162 (**Figures 1C–G**; **Figure S1**) IKE is an analog of erastin that inhibits system xC; ML162 is a potent inhibitor of GPX4. The HN12, HN8, and HN31 cell lines showed significant susceptibility to ferroptosis induced by either IKE or ML162 (**Figure S1**). The addition of the lipid anti-oxidant and ferroptosis inhibitor ferrostatin-1 rescues these cell lines from ferroptotic cell death (**Figures 1C–E**). The JHU-022 cell line showed moderate susceptibility to IKE at the highest concentration (3 μM) (**Figure S1**; **Figure 1F**). As with the iron chelators, the NOKs showed little to no decrease in cell viability in response to IKE or ML162.

To corroborate these data, we next looked at TFR1, FPN, and ferritin heavy chain (FTH1) protein levels in these cell lines to get a snapshot of iron homeostasis. Consistent with previously published data, the TFR1 levels were higher in the HN12, HN8, and HN31 cell lines when compared to a non-malignant control. This corresponded to the cells most susceptible to ferroptosis inducers. FPN was noticeably lower in the HN8 and HN31 lines as well. The JHU-022 line, which was most resistant to ferroptosis of the cancer cell lines tested, had the lowest levels of TFR1. The non-malignant NOK cell line had much lower TFR1 and high FPN levels. NOK cells had the highest levels of FTH1 and the HNSCC lines had much lower FTH1 levels. Taken together, these results indicate that the metastatic HNSCC (but not the non-malignant NOK cells) are sensitive to iron chelation and ferroptosis and may exhibit an iron dependent phenotype.

### FPN over-expression disrupts iron metabolism in HNSCC

**Figure 2:**
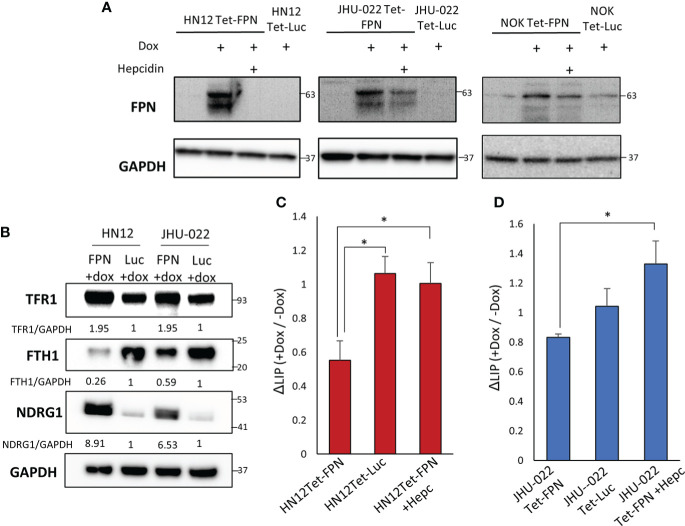
Stable Expression of FPN in the HN12, JHU-022, and NOK cell lines. **(A)** Western blot of FPN in the HN12-FPN/Luc, JHU-022-FPN/Luc, and NOK-FPN/Luc after treatment with 0.5 μg/mL of doxycycline for 48 hours. For rescue experiments, hepcidin was added to cell cultures with doxycycline at 20μM. **(B)** HN12-FPN/Luc and JHU-022-FPN/Luc were stimulated with 0.5 μg/mL dox for 72 hours and blotted for iron related proteins transferrin receptor 1 (TfR1), ferritin heavy chain 1 (FTH1), or N-myc downstream regulated protein (NDRG1). Quantification of TFR1, FTH1, and NDRG1 was performed using GAPDH as a loading control and normalized to the Luc control line. **(C) (D)** Measurement of the labile iron pool in cells using 1 μM FerroOrange. Changes in the levels of labile iron are represented as ratios of induced (+dox) samples normalized to un-induced (- dox) samples. (*P<0.05). All data is representative of 3 biological replicates.

To determine if changes in iron metabolism are a potential driver of HNSCC growth and proliferation or merely incidental we utilized an FPN over-expression system and assessed its effects on HNSCC cell lines and the non-malignant NOK line. The FPN CDS was cloned into the pLVX-TetOne-puro vector to create a conditional doxycycline-driven system to express FPN. The pLVX-Tet FPN and control vectors (pLVX-Tet Luc) were stably transduced into the HN12, JHU-022, and NOK cell lines (**Figure 2A**). To ensure that FPN was expressed on the cell membrane and that the phenotype observed was due to active FPN we treated the HNSCC cell lines with doxycycline and hepcidin. Hepcidin successfully knocked down the protein levels of FPN indicating that FPN was at the cell surface and active. In addition to this, immunofluorescence staining of the HN12-FPN/Luc expressing cells reveals FPN is expressed and distributed throughout the cell (**Figure S2D**).

FPN drives cellular iron efflux, thus FPN expression should lead to a decrease in labile iron levels. This should be reflected in the regulation of iron related genes. Consistent with this prediction, the HN12-FPN and JHU022-FPN lines had elevated levels of TfR1 and decreased levels of ferritin heavy chain 1 (FTH1) when compared to luciferase expressing controls (**Figure 2B**). NDRG1 (a gene commonly viewed as a metastasis suppressor) is elevated in iron deplete conditions (26). We found the levels of NDRG1 to be highly elevated in the FPN expressing lines (**Figure 2B**). To substantiate this, we directly measured the levels of labile iron using the FerroOrange dye. We found that labile iron levels were decreased in the FPN expressing lines compared to controls and that this decrease was reversed using hepcidin (**Figures 2C, D**).

### FPN expression inhibits HNSCC cell growth and proliferation

**Figure 3:**
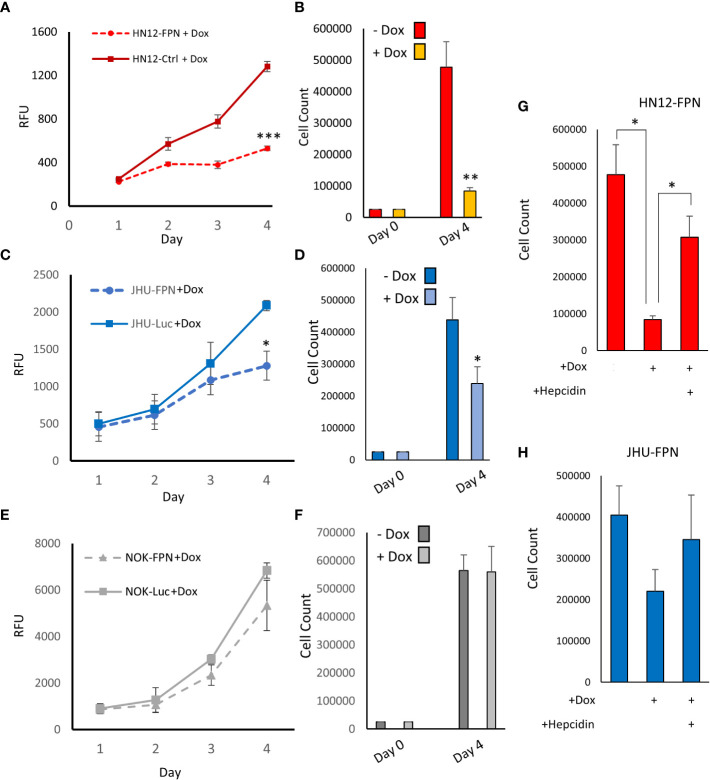
Expression of Ferroportin inhibits growth and proliferation of HNSCC. HN12 **(A)**, JHU-022 **(C)** and NOK **(E)** FPN expressing or Luc control expressing cells were seeded in 96 well plates at 1000 cells per well with 0.25 μg/mL dox and grown for 4 days. Growth was assessed using CellTiter-Blue reagent. HN12-FPN **(B)**, JHU-022-FPN **(D)**, and NOK-FPN **(F)** cells were seeded at 25,000 cells per well in a 6-well plate +/- 0.25 μg/mL dox. After 4 days cells were counted *via* hemocytometer. HN12-FPN **(G)** and JHU-FPN **(H)** were seeded in 6 well plates and treated with doxycycline (0.25 μg/mL), hepcidin (10 μM), or left untreated. Cells were grown for 4 days, after which cells were trypsinized and counted *via* hemocytometer. (*P<0.05; **P<0.01; ***P<0.001). All data is representative of 3 biological replicates.

**Figure 4:**
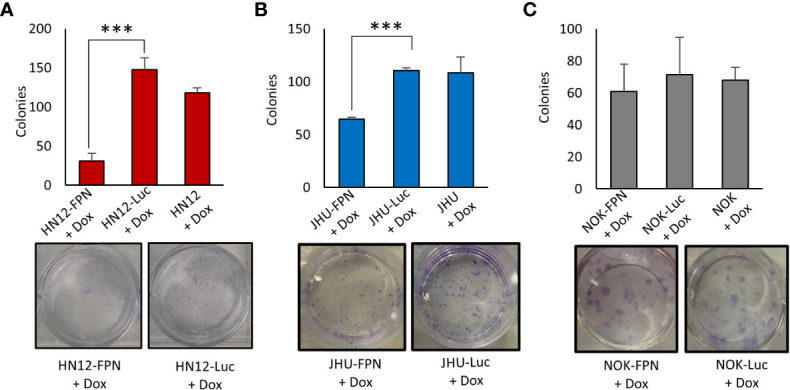
Expression of Ferroportin inhibits colony formation of HNSCC cell lines. Clonogenic assay quantification and a representative plate of HN12 **(A)**, JHU-022 **(B)**, and NOK **(C)** FPN expressing or Luc control expressing cells. Cells were seeded at 250 cells per well with 0.25 μg/mL dox and colonies were counted after 7-8 days. (***P<0.001) Data and images are representative of 3 biological replicates.

We found that expression of FPN caused a significant decrease in growth in the HNSCC cell lines (HN12 and JHU-022) when compared to non-expressing control cells when assessed *via* cell counts and Cell-titer blue assay (**Figures 3A–F**). After 4 days, the growth of the FPN expressing HNSCC cell lines were significantly lower than their luciferase expressing control lines. In the HN12 line specifically, significant differences in growth were seen after 48 hours, with little to no growth occurring after this time point (**Figure 3A**). The non-malignant NOK cell line showed no significant difference in growth between the FPN expressing line and luciferase control over 4 days (**Figure 3G, H**). There was no significant difference in the growth in cell lines in the absence of doxycycline and the parent cell lines (**Figure S2**). The addition of hepcidin to the HN12-FPN and JHU-022-FPN cultures rescued the cells from ferroportin mediated growth inhibitions (**Figures 3G–H**). These results were further confirmed using a clonogenic assay (**Figure 4**) Induction of FPN inhibited colony forming ability of the HNSCC cell lines (**Figures 4A, B**). Colony formation was not affected in the FPN expressing NOK cells (**Figure 4C**)

We have shown that the HN12 cell line is sensitive to ferroptosis induced by IKE treatment. Ferroptosis is dependent on elevated iron levels to induce lipid peroxide damage. Decreasing iron levels protect cells from ferroptotic cell death. Consistent with this, expression of ferroportin efficiently rescues the HN12 cell line from ferroptosis (**Figure S3**).

### FPN expression inhibits HNSCC cell cycle progression

**Figure 5:**
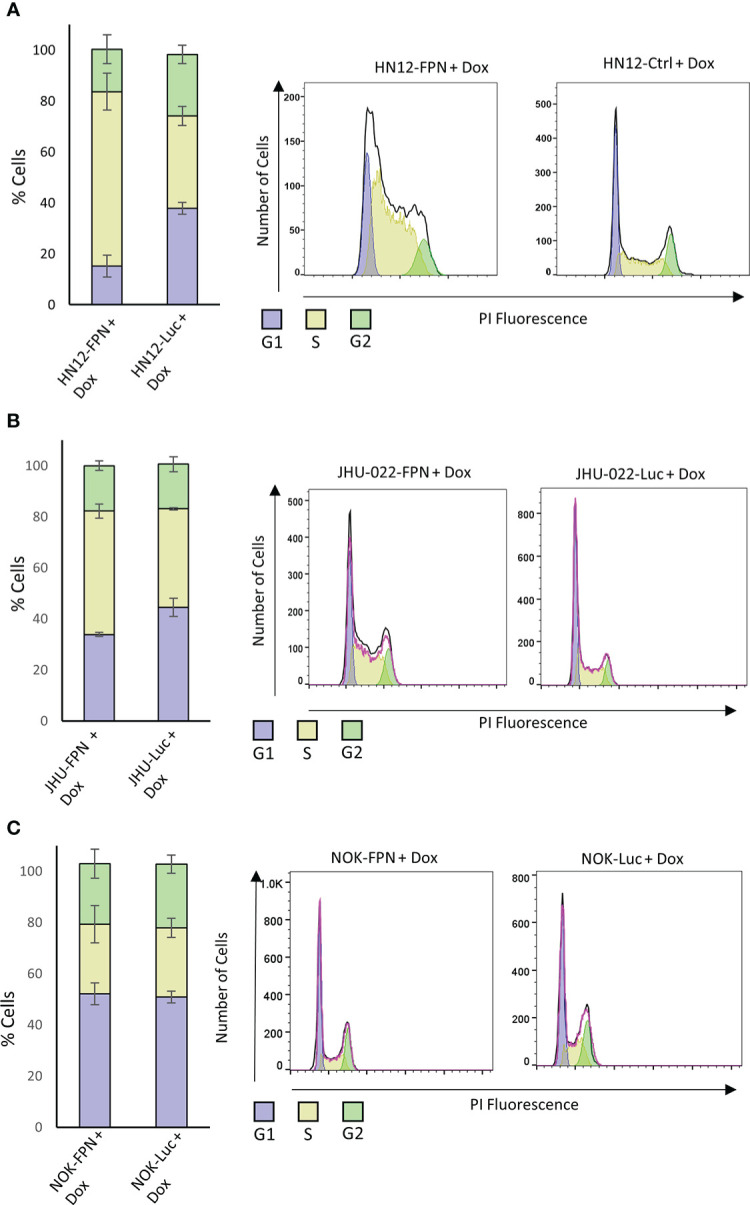
Ferroportin expression causes cell cycle arrest in HNSCC. FPN or Luciferase control expressing HN12 **(A)**, JHU-022 **(B)** and NOK **(C)** were grown with 0.5 μg/mL dox for 3 (HN12) or 4 days (NOK, JHU-022) and stained with propidium iodide to measure DNA content. The DNA content was assessed by flow cytometry and the cell cycle distribution was analyzed using FlowJo. All data is representative of 3 biological replicates.

Iron levels are linked to the progression of the cell cycle (27, 28). To determine the mechanisms by which FPN expression inhibits HNSCC growth and proliferation, we assessed the cell cycle of FPN expressing cell lines. As seen in **Figure 5**, FPN expression in HNSCC cell lines triggered cell cycle arrest in the S-phase. Specifically, most HN12-FPN cells were arrested in the S-phase (**Figure 5A**). The FPN expression in the JHU022 line yielded a modest arrest in the S-Phase (**Figure 5B**). FPN expression had no significant effect on the cell cycle in the NOK cell line (**Figure 5C**). Interestingly, this is not the case with cells treated with the iron chelator DFO (**Figure S4**). When treated with 50 uM DFO, HN12 cells are arrested in the G1 phase. This is consistent with previous studies: Iron chelation typically causes cell cycle arrest in the G1 phase. Thus, a different mechanism may be at play in the FPN inducing cell lines.

**Figure 6:**
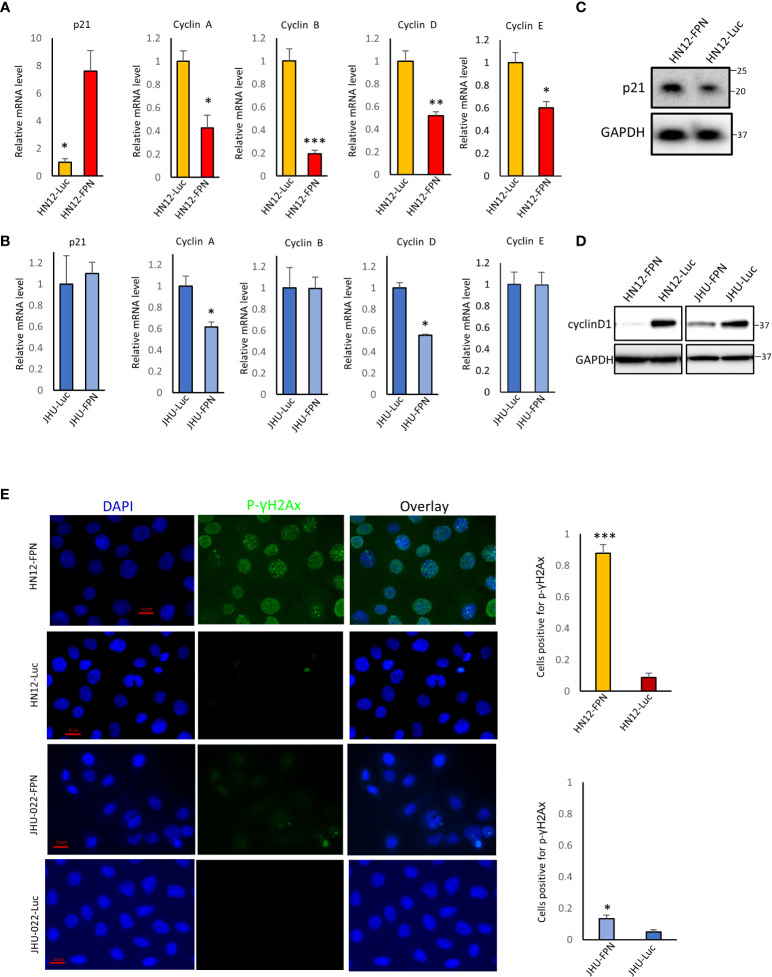
Ferroportin expression in HNSCC cell lines downregulates key cell cycle related genes **(A)**, **(B)** The mRNA levels of p21, cyclin A, cyclin B, cyclin D and cyclin E were assessed by qRT-PCR in HN12-FPN/Luc and JHU-022-FPN/Luc expressing cells grown in 0.5 μg/mL dox for 3 days. Levels of target genes are normalized to the levels of both GAPDH and beta-actin. Data is representative of 4 technical replicates performed in 3 biological replicates. **(C)** Western blot of p21 levels in HN12-FPN and HN12-Luc expressing cells. **(D)** Western blot of cyclinD1 levels in HN12-FPN/Luc and JHU-022/Luc expressing cells grown in 0.5 μg/mL dox for 3 days **(E)** HN12-FPN/Luc and JHU-022-FPN/Luc cells were stimulated with 0.5 μg/mL dox for 3 days and stained for p-γH2Ax (green) or DAPI (blue). Graphs represent the ratio of cells + for staining over the total number of quantified cells. Images are representative of 3 biological replicates. (*P<0.05; **P<0.01; ***P<0.001).

We next evaluated the effect of FPN expression on key cell cycle factors. The induction of p21 is a major S phase checkpoint pathway, thus we suspected that p21 may be responsible for the S phase arrest seen in the HNSCC cell lines. We found that the levels of p21 were significantly elevated in the HN12-FPN line at the RNA and protein level (**Figures 6A, B**) but not in the JHU-022-FPN line. (**Figure 6C**). Consistent with this, we found that the transcript levels of cyclins A, B, D, and E were all significantly decreased in the HN12-FPN line. In the JHU-022-FPN line, we found only Cyclin A and D were down-regulated (**Figure 6C**). Repression of cyclinD1 expression in FPN expressing HN12 and JHU-022 lines was confirmed *via* western blot (**Figure 6E**).

Since p21 is induced by DNA damage and cyclins are down-regulated by DNA damage, we asked whether FPN expression and iron depletion led to the accumulation of DNA damage. Phosphorylation of histone gamma H2AX is a recognized marker for DNA double strand breaks. As seen in **Figure 6D**, FPN overexpression led to a large increase in the levels of phospho(S139)-γH2AX in HN12-FPN cells. Comparatively, there was a modest increase in phospho(S139)-γH2AX in the JHU-022-FPN cells. The levels of phosopho(S139)-γH2AX in the parent HN12 and JHU-022 cell lines were similar to that of the luciferase expressing control cell lines, indicating loss of iron is causing DNA damage (**Figure S5**).

Notably, we also checked for apoptosis in our FPN expressing cell lines. We found that there was no significant population of cells in the sub-G1 phase, a marker of apoptosis (**Figure S6A**). Furthermore, caspase 3/7 activity was not significantly different in the FPN expressing HNSCC cells after 3 days of treatment with doxycycline (**Figure S6B**).

### FPN expression induces senescence in the HN12 cell line

**Figure 7:**
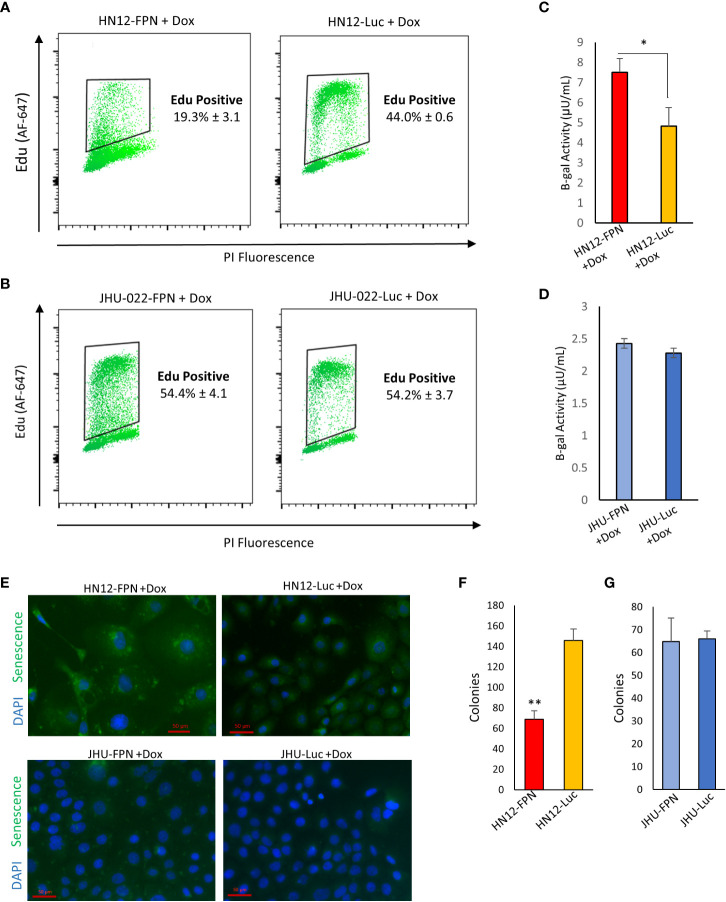
FPN expression induces senescence in HN12. **(A)**, **(B)** EDU incorporation of HN12-FPN/Luc **(A)** and JHU-022-FPN/Luc **(B)** grown with 0.5 μg/mL dox for 3 days. **(C, D)**. Beta-galactosidase activity of HN12-FPN/Luc **(C)** and JHU-022-FPN/Luc **(D)** after 7 days of exposure to 0.5 μg/mL dox. One μUnit is equal to 1 pmol of product generated per min. **(E)** HN12-FPN/Luc and JHU-022 FPN/Luc expressing cells were grown with 0.5 μg/mL dox for 37 days and stained for senescence using CellEvent Green Senescence Kit **(F)**, **(G)** Clonogenic assay of HN12-FPN/Luc and JHU-022-FPN/Luc expressing cells grown for 72 hours with 0.5 μg/mL after recovery in dox free media for 48 hours. (*P<0.05; **P<0.01).

Senescence is a well-documented response in cells experiencing catastrophic DNA damage. To determine whether FPN expression causes senescence in HNSCC we utilized an Edu incorporation assay to determine if DNA synthesis and replication are attenuated. FPN expression significantly reduced Edu incorporation in the HN12 cell line (**Figure 7A**). Loss of Edu incorporation is consistent with cells under a senescent phenotype. Despite seeing a modest increase in the S phase of the cell cycle in the JHU-022 cell line, FPN expression does not yield a significant change in the Edu incorporation during the experimental time frame (1.5 hours) (**Figure 7B**).

Senescence associated β-galactosidase activity is the most widely used biomarker to determine the state of a cell (29). To confirm that FPN expression is inducing senescence in the HN12 cell line and not the JHU-022 cell line, we measured the β-galactosidase activity. We found that the β-gal activity is significantly higher in the HN12-FPN induced cell line when compared to the Luc expressing control (**Figure 7C**). Consistent with previous data, there is no significant difference in the β-gal activity in the JHU-022 cell line (**Figure 7D**). This was further confirmed by staining for β-gal activity and assessing *via* fluorescence microscopy (**Figure 7E**). Notably, the levels of the β-gal activity are highest in the HN12-FPN expressing cells. Furthermore, the levels of β-gal activity are comparable in the parent HN12 and JHU-022 cell lines and the luciferase expressing controls. One of the most recognizable properties of senescence is the enlarged and flattened morphology of these cells. HN12 cells adopt a senescent cell morphology when FPN expression is induced (**Figure S7**).

To determine if FPN induced senescence was transient, HN12 and JHU-022 FPN and luciferase expressing lines were grown with doxycycline for 3 days. The doxycycline was removed and cells were washed and grown for additional 2 days with fresh doxycycline free media to recover. A clonogenic assay was performed on these cells. We found that the HN12-FPN cell line still had a significantly lower number of colonies recovered (**Figure 7E**) indicating the FPN mediated senescence was persistent. There was no difference in the colony numbers of the JHU-022-FPN and JHU-022-Luc lines after recovery in doxycycline free media (**Figure 7F**).

Finally, we wanted to test if deoxynucleotide depletion contributed to the senescent phenotype seen in the HN12-FPN cells. Studies have revealed that iron plays a crucial role in the maintenance of the dNTP pool as a component of ribonucleotide reductase (30–32). We supplemented HN12-FPN cells with deoxynucleotides and assessed their growth after 48 hours (**Figure S9**). We saw a partial rescue of the growth of these cells, however, over longer time courses this effect was not sustained.

### FPN expression inhibits growth of HNSCC tumors in an orthotopic xenograft model

**Figure 8:**
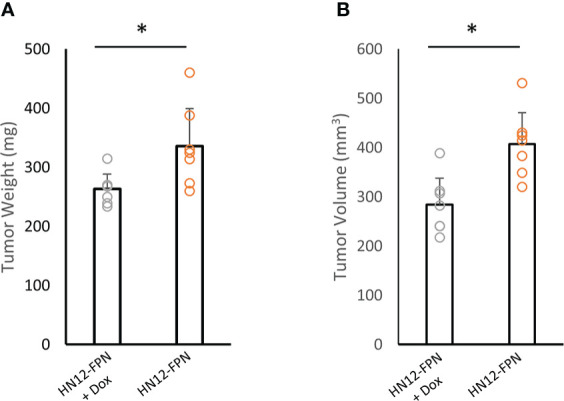
FPN expression inhibits growth of HN12 cells in an orthotopic xenograft model. HN12-FPN cells were implanted into the left cheek of mice and allowed to form tumors for 5 days. FPN expression was induced by adding 2mg/mL Doxycycline to the drinking water of mice. Control mice received normal drinking water. After 21 days, tumors were harvested and tumor weight **(A)** and tumor volume **(B)** were measured. For each group n=7 mice. (*P<0.05).

Given that FPN expression can induce cell cycle arrest and inhibit cancer cell proliferation, we utilized an oral orthotopic xenograft model to test the growth of the HN12 cell line *in vivo*. HN12-FPN cell line was implanted into the left cheek of NSG mice to establish tumors. Doxycycline was added to the drinking water to induce FPN expression in the experimental group and the control group was provided drinking water without doxycycline. In total, tumors were grown for 21 days before the termination of the experiment due to morbidity of mice in the control group. Tumors were harvested, and tumor volume and weight were assessed. Measurements indicate tumor weight and volume were significantly lower in the experimental group (FPN induced) when compared to the control group (**Figures 8A, B**). We assessed the levels of p21 in whole tumor lysates. Consistent with *in vitro* data, the levels of p21 were higher in tumors across the FPN expressing group when compared to the non-expressing control line (**Figure S10**). These results indicate the FPN expression suppresses the growth of HNSCC *in vivo*.

## Discussion

This is the first study to investigate the effect of FPN expression on oral keratinocytes and HNSCC. We show that FPN mediated iron depletion has a significant effect on growth inhibition, cell cycle arrest, and senescence. We saw two distinct responses in the HN12 and JHU-022 cell lines. The HN12 cell line exhibited a very iron “addicted” phenotype, where the loss of available iron led to near-total growth arrest, DNA damage, and senescence. This decrease in proliferation carried over into our *in vitro* orthotopic mouse model: tumors were significantly smaller in the FPN expressing xenografts. Notably in the HN12 cell line, growth arrest persisted despite the removal of doxycycline as assessed by clonogenic assays. The JHU-022 line saw a significant decrease in growth, however, this did not lead to high levels of DNA damage and the cells did not enter senescence. The induction of senescence is a time sensitive process and 7 days of doxycycline treatment may not be long enough to induce senescence. Notably, the senescence staining did indicate a slightly higher level of β-galactosidase activity in the JHU-FPN line when compared to the control cells (**Figure 7E**), however, this was not corroborated in the activity assay. Potentially longer incubations may be sufficient to induce senescence in the JHU-022 line.

One explanation for differences observed between the HN12 and JHU-022 cell lines in response to ferroportin mediated iron depletion is the reliance of the HN12 cells on the c-MYC oncogene. Amplification of MYC oncogenes has been demonstrated to cause sensitivity to ferroptosis and iron addiction in other cancer types (33, 34). Blotting for c-MYC levels in our panel of metastatic HNSCC cell lines reveals that the HN12 and HN8 cells have higher levels of c-MYC than the HN31 and JHU-022 cells (**Figure S11**). Coincidentally, HN12 and HN8 cells appear to be more sensitive to ferroptosis inducer IKE than HN31 and JHU-022 (**Figure 1**; **Figure S1**). Likewise, the sensitivity of HN12 cells to FPN mediated iron depletion (relative to the JHU-022 line) may be due an iron addicted phenotype caused by amplification of c-MYC. To investigate this link further, more studies probing the relationship of c-MYC and iron addiction in HNSCC is needed.

The addition of deoxynucleotides had a partial rescue effect on the HN12-FPN expressing cell line. Previous studies have shown that sufficient iron levels are extremely important for maintaining the available pool of deoxynucleotides needed for DNA synthesis and replication (32, 35). Lack of available dNTPs for DNA synthesis can lead to cell cycle arrest and DNA damage seen in HN12-FPN cells. A partial rescue was seen over 48 hours, however, the addition of deoxynucleotides alone was not able to fully rescue the cells from growth inhibition, implying that elevated iron levels may be playing a larger role than maintenance of the dNTP pool in these cells.

FPN expression had little to no effect on the non-malignant NOK cells. This was not unexpected, as the NOKs were resistant to growth inhibition by DFO and cell death *via* ferroptosis at the concentrations used in this study. Expression of FPN caused significant changes in metabolism in the HNSCC cell lines. Iron homeostasis is regulated by a sensitive feedback system. When cells are depleted of iron, we saw levels of TFR1 go up and levels of FTH1 go down to compensate for the lack of available iron, validating our experimental model.

It should be noted that we made many attempts at creating a stable expression of FPN in the HN8 and HN31 cells, yet we were unsuccessful. When we were able to see FPN expression, the elevated levels were quickly lost after a passage, even after isolation of a monoclonal culture. Notably, these two cell lines have the lowest levels of FPN of the cells we tested. There appears to be a significant selective pressure against FPN expression in these cell lines.

We chose to use a model of FPN expression to simulate iron depletion rather than the normal iron chelation strategies in wide use. FPN is unable to liberate bound iron from non-physiological targets. DFO, and other iron chelators, are capable of stripping iron from bound proteins and storage molecules due to their extremely high affinity for iron (13, 36). Iron chelators are well known for their use in exploring the biological mechanisms of iron homeostasis and their therapeutic benefits in iron related pathologies. However, there are noted downsides to iron chelation therapy, which are prone to off target side effects (37). Furthermore, their ability to chelate intra-cellular iron has brought into question if they truly mimic physiologically relevant iron deplete conditions (38). Ligands with appropriate reduction potentials and high affinity for Fe(II) can force reduction and release of Tf-bound Fe(III) that would not be seen under physiological circumstances (39). Furthermore, binding of transferrin to TFR1 and TFR2 causes them to act as an important signal and binding partner on the cell membrane (such as with HFE or EGFR) (40). Thus our strategy of FPN expression more resembles an iron starvation phenotype in an iron deplete environment.

Still, there are similarities in the response of these cells to iron chelator treatment and FPN over-expression. The inhibition of cell proliferation, induction of DNA damage, and cell cycle arrest have been observed in DFO treatment. There are also differences. Treatment of cells with DFO causes cells to arrest in G1 phase, *via* the action of IRP2 (41). In our study, we saw a decrease in the population of cells in G_0_/G_1_ indicating the mechanisms between iron chelation treatment and FPN mediated iron depletion may be different.

Iron is a necessary nutrient for cellular health however, not every cell has the same need for iron. In this study we reveal 3 very different responses to FPN mediated iron depletion. HN12 cells are almost completely incapable of growing without elevated iron levels and enter a senescent state. Even after the removal of doxycycline and recovery, their growth is still arrested (**Figure 7F**). JHU-022 cells grow at a decreased rate, however, they do not enter senescence over the time frames used in these studies and growth resumes immediately after removal of FPN induction (**Figure 7G**). NOK cells are unperturbed by increased FPN expression – they may be able to compensate for this lack of iron in other ways. This implies that different malignant cell lines have different requirements for iron supplementation. What drives this difference, and how this may be exploited for therapeutic gain is an active area of research.

## Materials and methods

### Cell culture

The HN12, HN31, and HN8 cell lines were cultured in DMEM + 10% FBS. The JHU-022 cell line was cultured in DMEM/F12 + 10% FBS. These cell lines are from lymph node metastasis derived from the oral cavity and are HPV-negative (42, 43). TERT-Immortalized Normal Oral Keratinocytes (NOKs) were cultured in keratinocyte medium (Sciencell). Cells were grown in a humidified atmosphere of 5% CO_2_ at 37°C. All cell lines were authenticated *via* Short Tandem repeat profiling (ATCC). TERT-Immortalized NOKs were a gift from the lab of Michael McVoy. Cells were screened for mycoplasma using PCR or MycoStrip™ mycoplasma detection kit (*Invivo*gen).

### Viral packaging infection and preparation of FPN expressing cell lines

The tet-inducible lentivirus vectors pLVX-TetOne-puro vector and pLVX-Tetone-puro-Lucif control vector were purchased from Takara Bio. The FPN protein coding sequence was amplified *via* PCR to have sequences complementary to the pLVX-TetOne vector on the 5’ and 3’ termini. The pLVX-TetOne-puro vector was digested with *BamHI* and *EcoRI* and combined with the FPN CDS using NEB HiFi DNA assembly master mix (New England Biolabs) to create the pLVX-tet-FPN vector. Clones were screened and confirmed *via* sequencing.

Viral particles were created *via* co-transfection of the pLVX-tet-FPN vector and 4^th^ generation packaging vectors using the Lenti-X Packaging Single Shots (VSV-G) (Takara Bio) in Lenti-X HEK 293T cells. Viral particles for the luciferase control plasmid were created similarly. Two days after transfection, viral particles were collected from the supernatant and filtered with a 0.45 μm filter. The HN12, JHU-022, and NOK cell lines were transduced with viral particles in the presence of polybrene (10 μg/mL) at ~50-70% confluency. After 48 hours, cells were passaged, and stable clonal populations were selected using puromycin.

### Cell growth

Cell growth and proliferation were measured by cell counting or CellTiter blue assay (Promega). For cell counting, cells were seeded in 6-well plates at 25,000 cells per well and allowed to grow for 4 days. After incubation, cells were harvested and counted using a hemocytometer. For growth curves, cells were seeded in a clear-bottom, black 96-well plate at 1000 cells per well. Cell growth was assessed at 24, 48, 72, and 96 hours using 10 μL CellTiter-blue reagent and measuring fluorescence (560nm Ex/590nm Em) after 1 hour.

### Cell viability/survival assays

CellTiter-Glo assays were performed by seeding cells at 3-5k cells per well (such that cells were at least 30% confluency at the time of treatment) in a black 96-well plate. Supplemental compounds were added at the desired final concentration the next day and incubated for 3 days. After incubation, 25 μL of CellTiter-Glo reagent was added to each well and measured on an H1 BioTek plate reader as per manufacturer’s protocol. Non-treated cells seeded in parallel were used to determine total cell growth.

Cell survival was assessed *via* flow cytometry utilizing 7-AAD staining. Cells were seeded in 6 well plates and compounds were added at the final concentration the next day and incubated for 3 days. Cells were trypsinized and combined with cell culture supernatant. Cells were pelleted and resuspended in PBS + 10% FBS and stained with 7-AAD (ThermoFisher). 7-AAD staining was assessed using a BD FACSCanto II cell cytometer with 488nm excitation and emission was collected using a 670LP filter. Data was analyzed using BD FACSDiva 8.0.1 software.

### Clonogenic assays

Cells were harvested and seeded in a 12-well plate at 250 cells per well. Doxycycline was added at a final concentration of 0.5 μg/mL. Plates were incubated for 7-10 days (until colonies >~50 cells began to form). Afterward, cells were washed and fixed using methanol. To each well, 0.1% crystal violet was added and wells were washed and colonies were counted.

To determine if FPN mediated inhibition of cell growth was transient or permanent, FPN-induced and control cells were grown for 3 days with doxycycline. Cells were then washed and doxycycline free media was added and cells were grown for an additional 2 days. After incubation, cells were trypsinized and seeded at 250 cells per well in a 12-well plate and incubated for 7-10 days. Colonies were then counted as described above.

### Western blot

Cells were lysed in RIPA lysis buffer (150 mM NaCl, 1% TritonX-100, 0.5% Deoxycholic acid, 0.1% SDS, 50 mM Tris pH 7.4) plus protease inhibitor cocktail (Millipore-Sigma). For whole tumor lysates, harvested tumors were frozen in liquid nitrogen and stored. Frozen tumors were thawed on ice and cut into fragments using a scalpel. RIPA buffer was added to fragments and samples were homogenized using a pestle in the tube. Samples were sonicated and incubated on ice for 20 minutes before being spun down to clarify the lysate. Samples were separated on 4-12% Bis-Tris PAGE gels and transferred to PVDF membranes set in NuPAGE transfer buffer (Thermo-Fisher) containing 20% methanol and blocked using 5% milk in TBST. Membranes were probed with primary and secondary antibodies and detected using chemiluminescence in SynGene G-Box. Antibodies used: GAPDH (Novus Bio - #NB100-56875), FPN (Novus Bio - #NBP1-21502), TFR1 (ThermoFisher - #13-6800), FTH1 (Cell Signaling Technology - #4393), NDGRG1 (Cell Signaling Technology - #9485), p21 (Cell Signaling Technology - #2947), cyclinD1 (Cell Signaling Technology - #55506).

### Real time qRT-PCR

The RNeasy kit (Qiagen) was used to purify RNA from cells. Residual DNA was removed using the DNA-free DNAse kit (ThermoFisher). The cDNA was generated using the High-Capacity cDNA Reverse transcriptase kit (ThermoFisher). Real Time qRT-PCR was performed using SYBR-green based detection on a Quant Studio 3 real-time PCR system using PowerUP Syber green MasterMix (ThermoFisher). Primers for GAPDH and beta-actin were used as endogenous controls to which RNA levels of target genes were normalized to. Primers used for qRT-PCR analysis are listed in **Supplementary Table S1**.

### Labile iron pool assay

Labile iron levels were measured using the FerroOrange labile ferrous iron detecting probe (Goryo Chemical Inc). Cells were grown in a 6-well plate for 72 hours plus/minus doxycycline. Cells were then seeded in a black, clear bottom, 96-well plate at 5000 cells per well. The next day, cells were washed with serum-free media containing 50 μM DFO and twice with HBSS to remove free extra-cellular iron. Cells were then incubated with 1μM of probe in HBSS for 30 minutes at 37°C. After incubation, the probe was removed from the cells and fresh HBSS was added, and fluorescence (Ex 542, Em 572) was measured. Values were normalized to the amount of total cells as measured by CellTiter-Glo assay.

### Cell cycle analysis

Cells were grown with doxycycline for 3 days (HN12) or 4 days (JHU-022, NOKs) to induce FPN expression. Cells were harvested by trypsinization and washed with PBS and resuspended in 300 μL of PBS and fixed *via* drop wise addition of -20°C absolute ethanol to a final concentration of 70% and incubated at -20°C for at least 1 hour. Fixed cells were washed with PBS and resuspended with FxCycle PI/RNase staining solution (ThermoFisher #F10797). Cells were incubated for at least 30 minutes at room temperature. Fluorescence was measured on a BD FACSCanto II cell cytometer using 488nm excitation and emission was collected using a 670LP filter. FlowJo v10.8.1 was utilized to perform cell cycle calculations.

### Edu incorporation

Edu incorporation was measured using the Click-it Plus Edu Alexa Fluor 647 Flow Cytometry Assay Kit (ThermoFisher C10634). Cells were grown for 3 days with doxycycline to induce trans gene expression and were then incubated with 20 μM Edu for 1.5 hours. After Edu incubations, cells were washed and trypsinized. Incorporated Edu was labeled as per the manufacturer’s protocol. After labeling Edu, total nuclear DNA content was stained using FxCycle PI/RNase solution. Fluorescence was measured on a BD FACSCanto II cell cytometer using 633 excitation and emission was collected using a 660nm/20BP filter. PI fluorescence was measured as detailed above. FlowJo v10.8.1 was utilized to perform Edu incorporation calculations.

### Caspase activity assay

The Caspase-3/7-Glo assay was utilized to measure caspase activity. Cells were grown for 3 days with doxycycline and then plated in 96 well plates at 10,000 cells per well. The next day, Caspase-3/7-Glo reagent was added to each well and incubated for 1 hour at room temperature. Luminescence was measured using an H1 BioTek plate reader.

### Immunofluorescence staining of cells

Cells were passaged into 4 chamber slides (Millipore-Sigma) and grown with doxycycline for 3 days. Cells were washed and then fixed and permeabilized using methanol before blocking with 1% BSA in PBS for 1 hour. Cells were incubated overnight with anti-human FPN antibody (Novus Bio - #NBP1-21502) in blocking buffer. Anti-Rabbit DyLight 488 secondary antibody (Novus Bio - #NBP1-72944) was used to stain cells. Slides were mounted using Prolong Gold anti-fade reagent plus DAPI (ThermoFisher) and imaged on a on a Keyence BZ-X810 microscope

### Beta-galactosidase activity assay and senescence staining

The beta-galactosidase activity assay was purchased from Abcam (Ab287846). Cells to be tested for activity were grown in a 6-well plate for 7 days in the presence of doxycycline. For input, 500,000 cells were used and the protocol was followed per the manufacturer’s instructions. The reaction was performed at room temperature in a 96-well plate and read using an H1 BioTek plate reader.

The CellEvent Senescence Green Detection Kit (ThermoFisher) was utilized to stain cells for senescent associated beta-galactosidase activity. Cells were grown for 7 days in the presence of doxycycline. Cells were then seeded in 4 chamber slides (Millipore-Sigma) and incubated overnight. Cells were fixed with 2% paraformaldehyde for 10 minutes, washed, and incubated with the CellEvent reagent for 2.5 hours at 37°C (without CO_2_). Cells were washed, mounted using Prolong Anti-fade with DAPI, and imaged on a Keyence BZ-X810 microscope.

### Phospho-(S139) gamma-H2Ax immunofluorescence

Cells were seeded into a 4-chamber slide (Millipore-Sigma) and grown for 72 hours in 0.5 μg/mL doxycycline. Cells were fixed in 4% paraformaldehyde for 10 minutes and the cell membrane was permeabilized using 90% methanol for 10 minutes. Slides were blocked using 1% BSA and incubated using anti-p-γH2Ax antibody (Abcam #242296) for 1 hour at room temp then stained using FITC conjugated secondary antibody. Coverslips were mounted using Prolong Anti-fade with DAPI (ThermoFisher) and imaged on a Keyence BZ-X810 microscope.

For calculations, staining was repeated three times. For each biological replicate, 100-150 random cells were imaged and counted and cells positive for p-γH2Ax staining were quantified against the total amount of cells.

### Mouse xenograft studies

All animal procedures were approved by the VCU Institutional Animal Care and Use Committee (IACUC) under protocol AD10002926. Six week old female NSG (NOD-*scid* IL2Rgamma^null^) mice were purchased from the VCU Massey Cancer Mouse Models Core. To each mouse, 50,000 HN12-FPN cells in serum-free media were injected into the left cheek in a 1:1 mixture of cells to Matrigel. Five days after injection mice were randomized into two groups. FPN expression was induced in mouse xenografts by adding 2mg/mL of doxycycline to the drinking water of one group. The control group received normal drinking water. Seven mice for each group (plus/minus doxycycline) were used for a total of n=14 mice. After 21 days, mice were sacrificed, tumor volume was recorded, and tumors were weighed. No surviving mice were excluded from the experiment. Differences in groups were calculated using a one-tailed T-test of equal variances.

### Statistical analysis

All *in vitro* experiments were performed at least 3 times. Statistical analysis was performed in excel. Comparison tests were performed using a two-tailed unpaired T-test of equal variance. Results are reported as mean plus/minus the standard deviation of the mean.

## Data availability statement

The raw data supporting the conclusions of this article will be made available by the authors, without undue reservation.

## Ethics statement

The animal study was reviewed and approved by VCU Institutional Animal Care and Use Committee (IACUC); protocol AD10002926.

## Author contributions

**BB:** Conceptualization, Investigation, Data Curation, Formal Analysis, Funding Acquisition, Methodology, Visualization, Writing – Original Draft, Writing – Review and Editing. **JL:** Conceptualization, Supervision, Project Administration, Funding Acquisition, Methodology, Writing – Review and Editing. All authors contributed to the article and approved the submitted version.
