# Supplementary material for: Ferroportin depletes iron needed for cell cycle progression in head and neck squamous cell carcinoma
Source: Front Oncol. 2023 Jan 9;12:1025434. doi: 10.3389/fonc.2022.1025434 (PMC9868905; doi:10.3389/fonc.2022.1025434)
Supplement: Supplementary file 1 [file DataSheet_1.pdf]

Supplementals

Figure S1

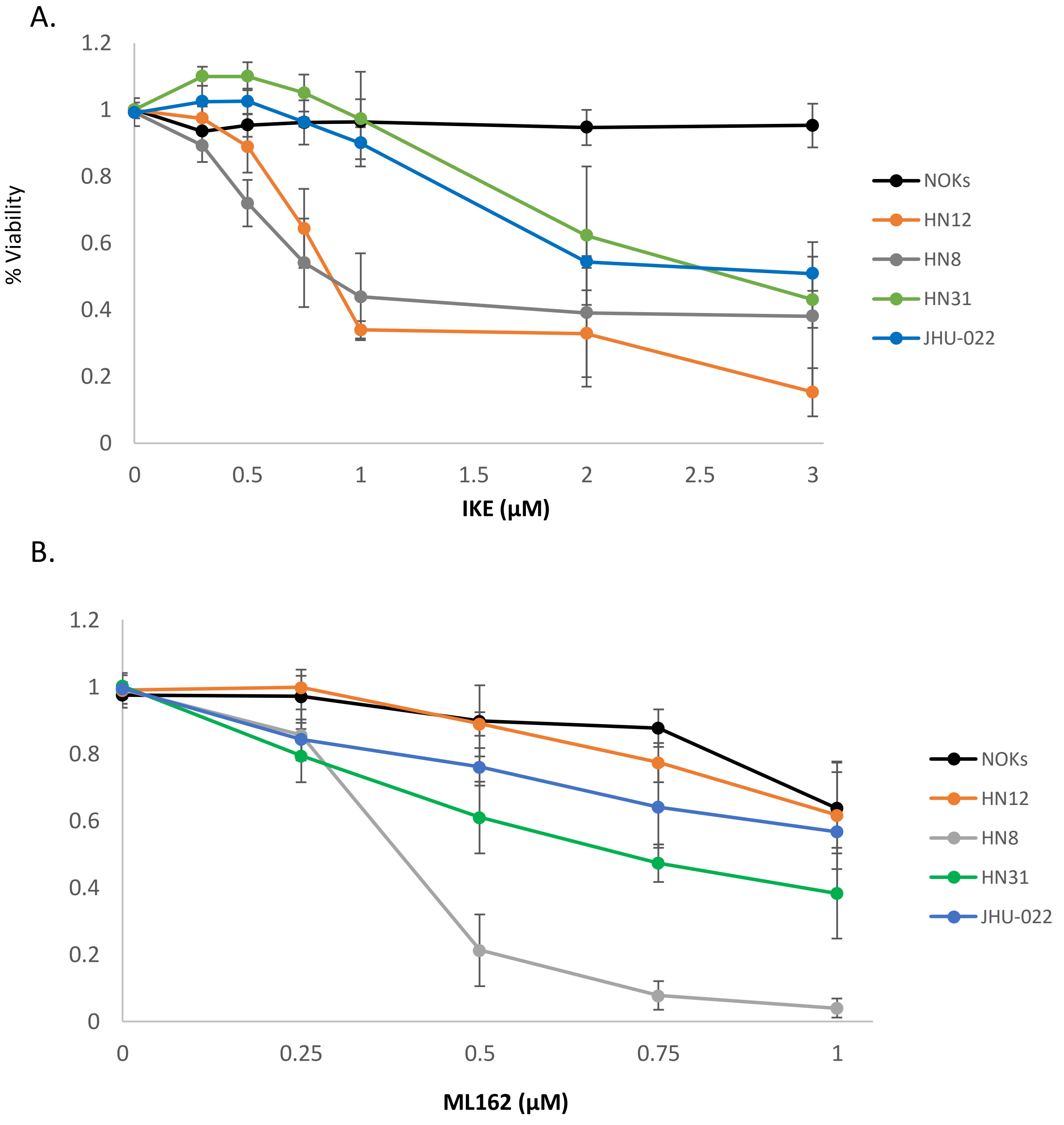

Figure S1 – HNSCC cell lines are sensitive to ferroptosis inducers. Cells were treated with increasing concentrations (**A.**) Imidazole Ketone Erastin (IKE) or (**B.**) ML162 and cell survival was assessed by CellTiter-glo. Cells were treated for 72 hours with the indicated concentrations of drugs.

Figure S2

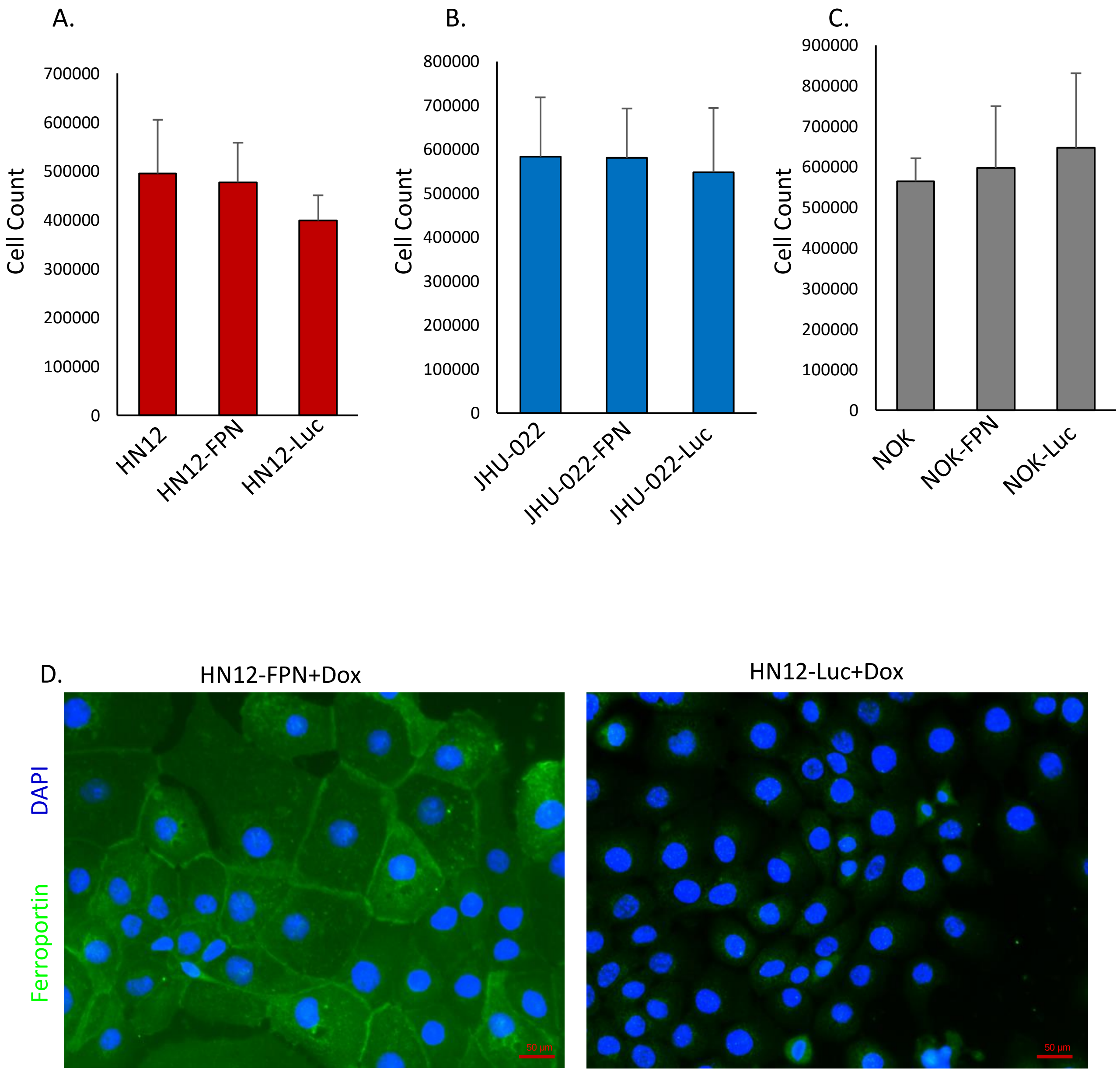

Figure S2 – There is no significant difference in the growth of the HN12 (**A.**), JHU-022 (**B.**) or NOK (**C.**) wild-type cell lines and their FPN and Luciferase expressing derived cell lines in the absences of doxycycline. **D.** Immunofluorescecne staining of FPN in the HN12-FPN and HN12-Luc cells grown with 0.5 µg/mL doxycycline for 3 days. FPN is stained in green and nuclei are stained in blue.

Figure S3

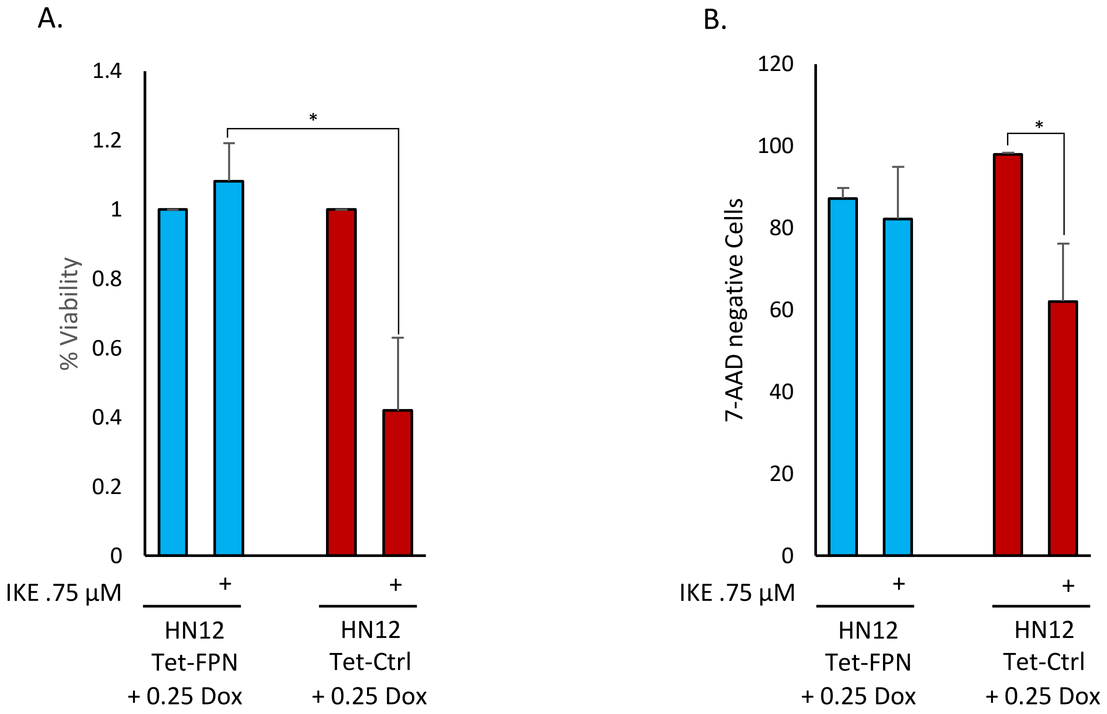

Figure S3 – A. HN12-FPN cells or HN12-Luc cells were seeded in a 96 well plate +/- doxycycline (0.25 µg/mL). The next day IKE (0.75 µM) was added and cells were treated for 3 days. Cell viability was assessed via CellTiter-glo assay. IKE treated conditions were normalized to untreated conditions plus doxycycline. **B.** HN12-FPN and HN12-Luc cells were seeded 6 well plates plus 0.25 µg/mL doxycycline. The next day 0.75 µM IKE was added ad cells were incubated for 3 days. After incubation cells were trypsinized and stained with 7-AAD to assess cell survival. Results were analyzed via flow cytometry.

Figure S4

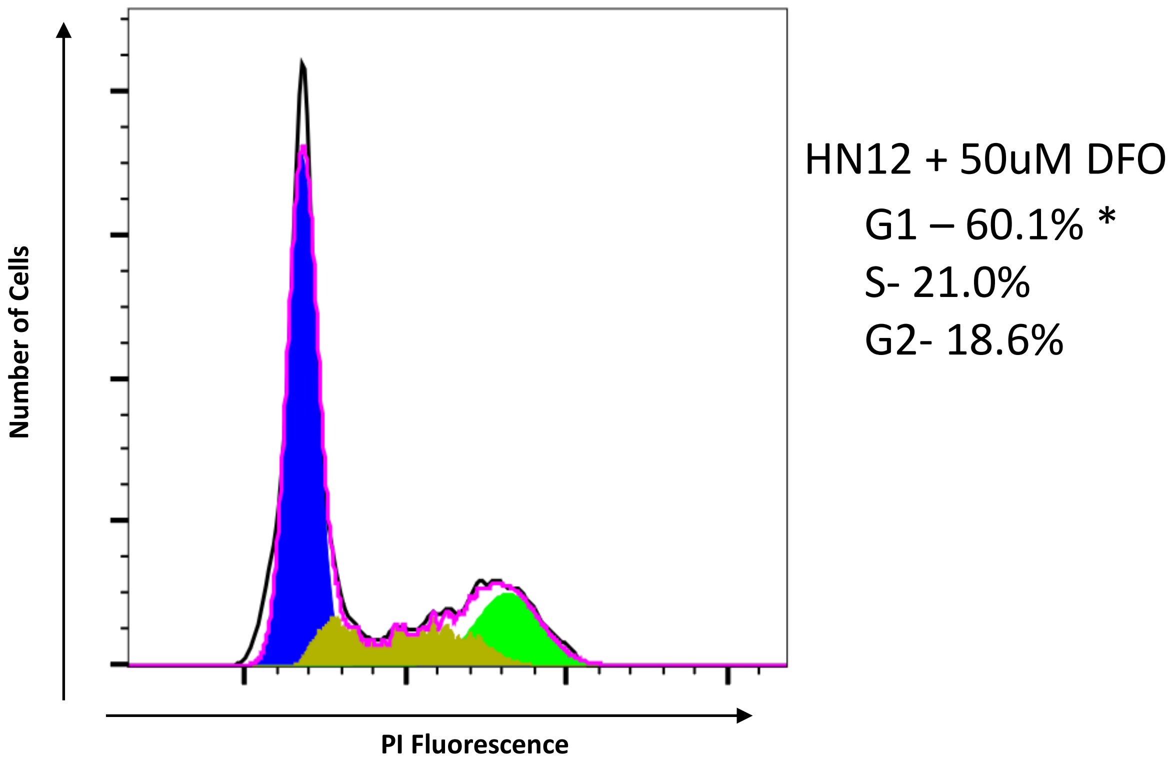

Figure S4 – DFO arrests cells in G_0/G_1 phase. HN12 cells were treated with 50 µM for 72 hours. Cells then stained with propidium iodide to measure DNA content and measured using flow cytometry.

Figure S5

A.

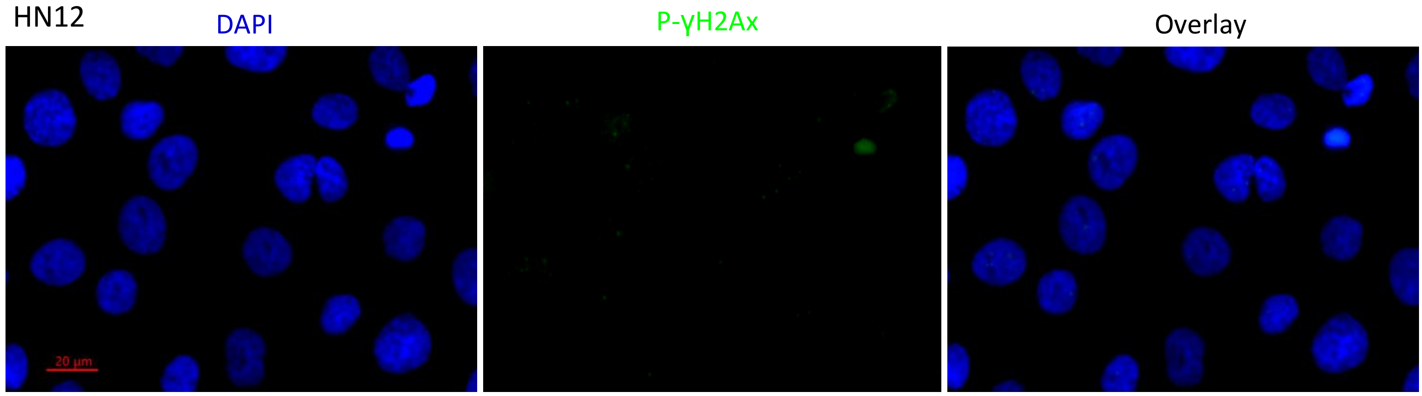

B.

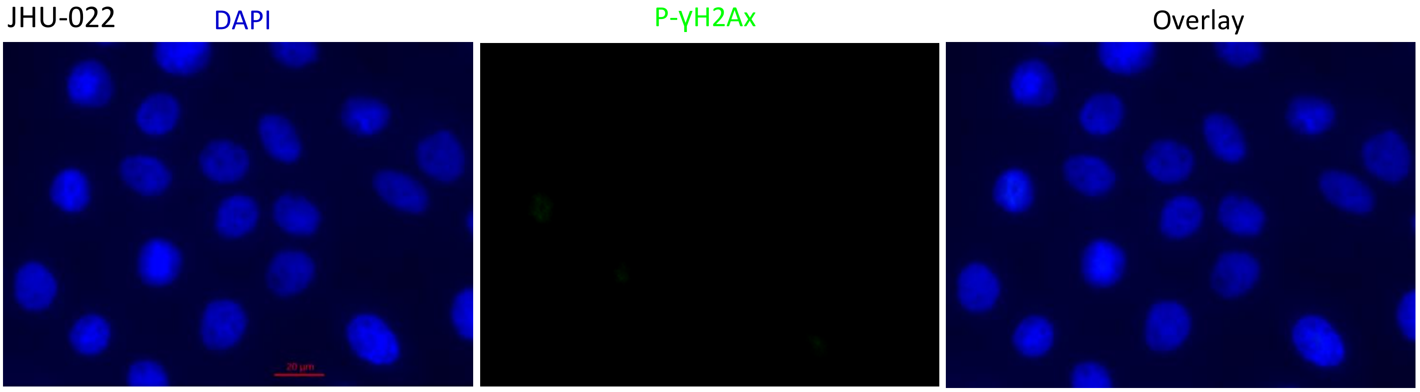

Figure S5 – Levels of p-γH2Ax staining in the wild-type HN12 (**A.**) and JHU-022 (**B.**) cell lines.

Figure S6

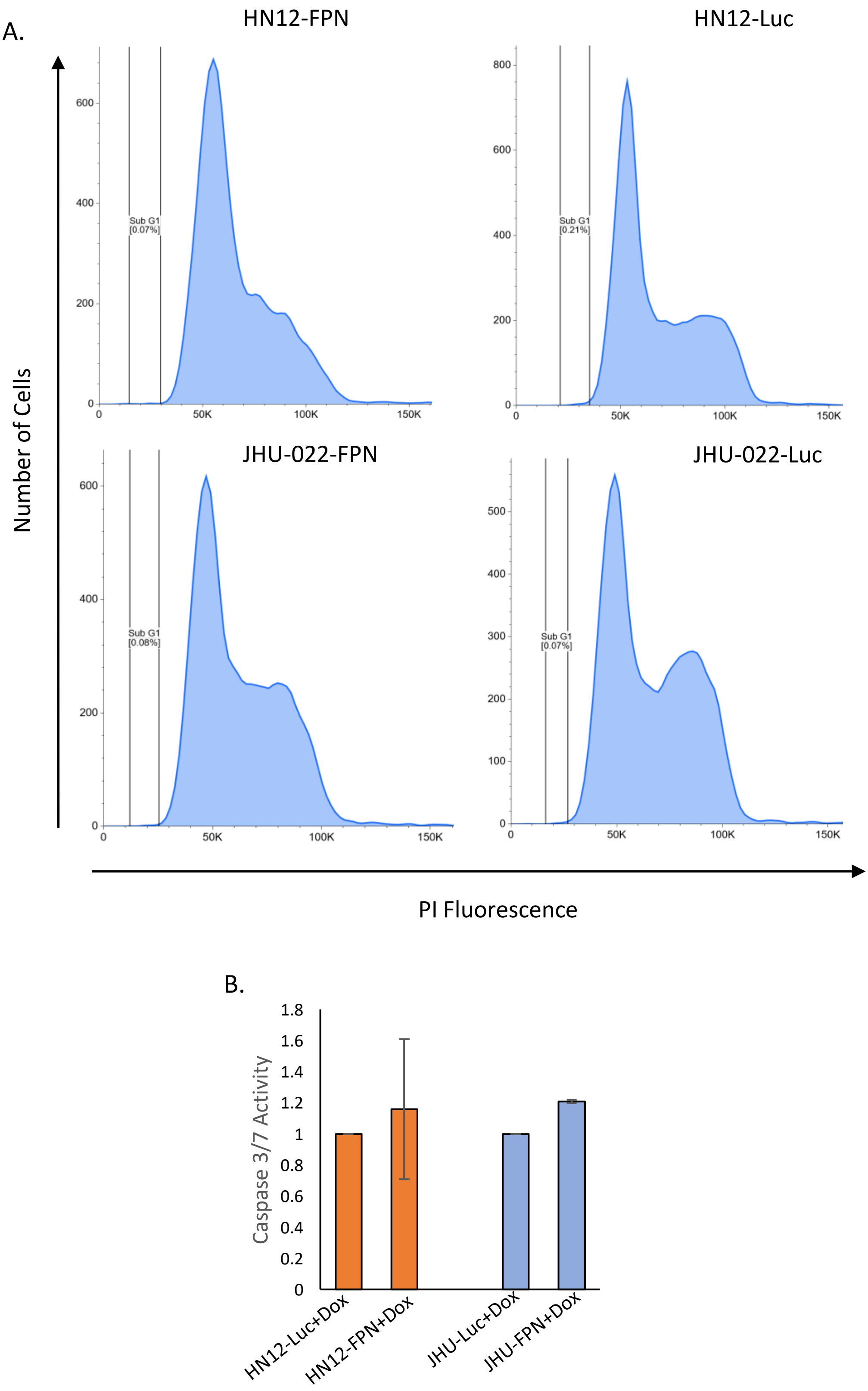
A.
HN12-FPN
HN12-Luc
JHU-022-FPN
JHU-022-Luc
Number of Cells
PI Fluorescence
Sub G1 [0.07%]
Sub G1 [0.21%]
Sub G1 [0.08%]
Sub G1 [0.07%]
B.
Caspase 3/7 Activity
HN12-Luc+Dox
HN12-FPN+Dox
JHU-Luc+Dox
JHU-FPN+Dox

Figure S6 – FPN over-expression does not cause apoptosis in HN12 and JHU-022 cells. **A.** Sub-G1 analysis of HN12-FPN/Luc and JHU-022-FPN/Luc cells after 3 (HN12) or 4 (JHU-022) incubation with 0.5 µg/mL doxycycline. **B.** Caspase 3/7 activity assessed via Caspase3/7-Glo assay of HN12-FPN/Luc and JHU-022 FPN/Luc after 3 days growth with 0.5 µg/mL doxycycline.

Figure S7

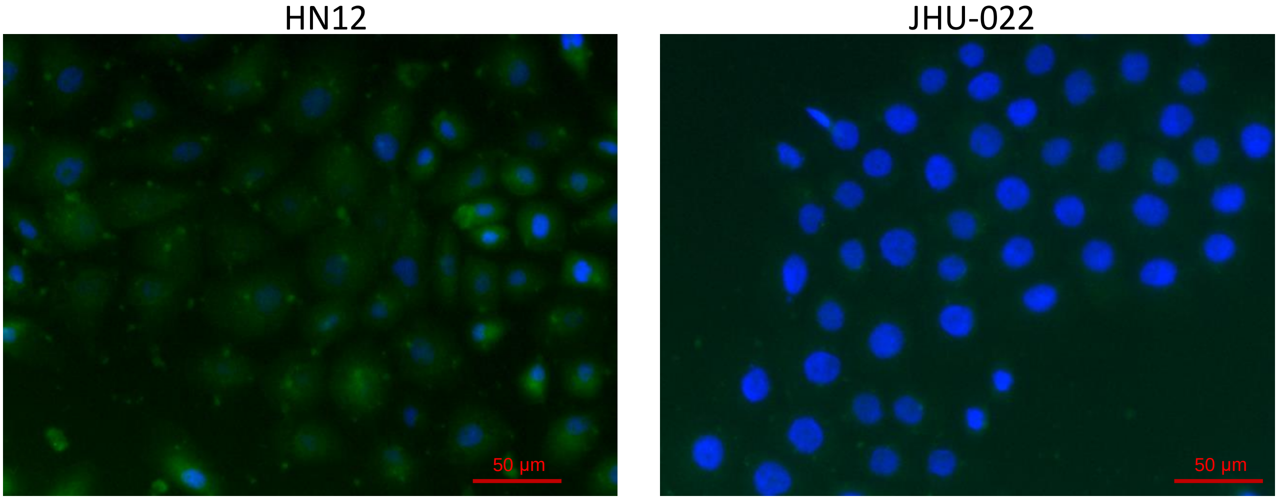

Figure S7 – Levels of β-galactosidase activity in the wildtype HN12 and JHU-022 cell lines assessed using the CellEvent Green Senescence Kit.

Figure S8

A. B.

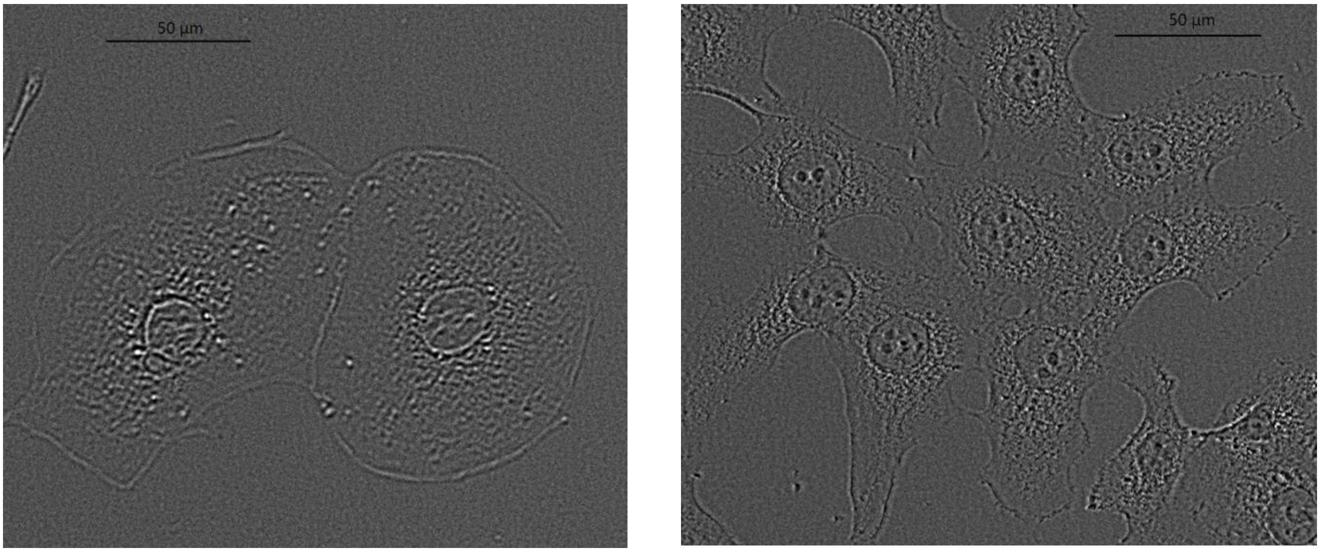

Figure S8- HN12-FPN cells have typical senescent cell morphology. Phase contrast images of HN12-FPN (A.) and HN12-Luc (B.)cells stained for γH2Ax.

Figure S9

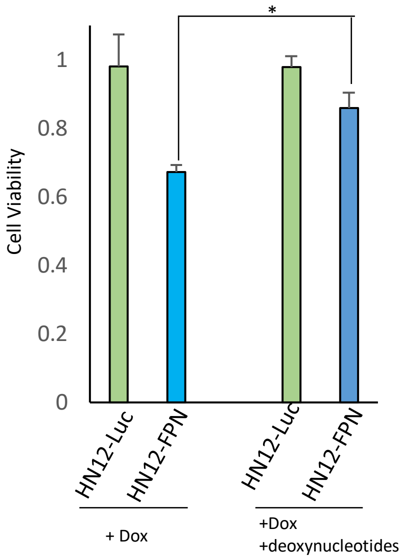

Figure S9 – The addition of deoxynucleotides partially rescues cells from growth inhibition. HN12-FPN and HN12-Luc cells were seeded in 96 well plates and grown with 0.25 µg/mL of doxycycline. Cultures were supplemented with 10 µg/mL of 2’-deoxyadenosine, 2’-deoxycytidine, 2’-deoxyguanidine, and thymidine where indicated. Cells were cultured for 48 hours and growth was assessed using Celltiter-blue assay. Growth was normalized to cells untreated with doxycycline.

Figure S10

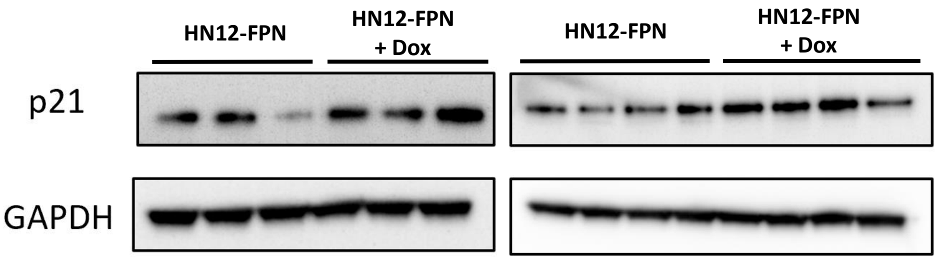

Figure S10 – Western blot for p21 from HN12-FPN (control) and HN12-FPN + dox whole tumor lysates from orthotopic xenograft tumors in NSG mice.

Figure S11

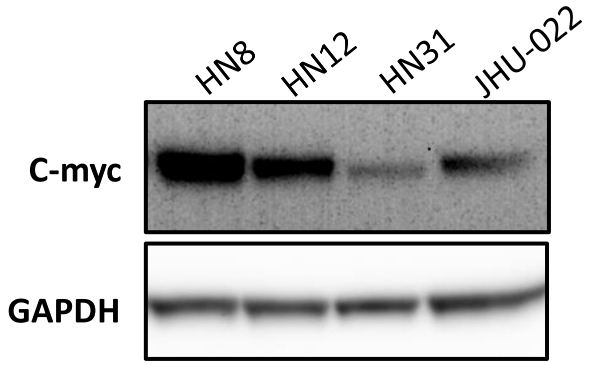

Figure S11 – Western blot for c-MYC expression in HN12, HN8, HN31, and JHU-022 cell lines.
