# Supplementary material for: Ferroportin depletes iron needed for cell cycle progression in head and neck squamous cell carcinoma
Source: Front Oncol. 2023 Jan 9;12:1025434. doi: 10.3389/fonc.2022.1025434 (PMC9868905; doi:10.3389/fonc.2022.1025434)
Supplement: Supplementary file 2 [file DataSheet_2.pdf]

Primer Name	Primer Sequence
p21-qPCR-F	CGC TCT ACA TCT TCT GCC TTA GTC
p21-qPCR-R	GAA CCT CTC ATT CAA CCG CCT AG
CyclinA-qPCR-F	TGG TTA GTT GAA GTA GGA GAA G
CyclinA-qPCR-R	TTG GTG TAG GTA TCA TCT GTA ATG
CyclinB-qPCR-F	TGG TTG ATA CTG CCT CTC C
CyclinB-qPCR-R	TCT GAC TGC TTG CTC TTC C
CyclinD-qPCR-F	TGA ACT ACC TGG ACC GCT TC
CyclinD-qPCR-R	AGC TTG TTC ACC AGG AGC AG
CyclinE-qPCR-F	GTT ATA AGG GAG ACG GGG AG
CyclinE-qPCR-R	TGC TCT GCT TCT TAC CGC TC
B-actin-qPCR-F	TTG CCG ACA GGA TGC AGA AGG A
B-actin-qPCR-R	AGG TGG ACA GCG AGG CCA GGA
GAPDH-qPCR-F	TGG TAT CGT GGA AGG ACT CAT GAC
GAPDH-qPCR-R	ATG CCA GTG AGC TTC CCG TTC AGC
FPN-pLVX-Tetone-F	CCC TCG TAA AGA ATT ATG ACC AGG GCG GGA GAT CAC AAC
FPN-pLVX-Tetone-R	GAG GTG GTC TGG ATC TCA AAC AAC AGA TGT ATT TGC TTG
